# Variability in spine radiosurgery treatment planning – results of an international multi-institutional study

**DOI:** 10.1186/s13014-016-0631-9

**Published:** 2016-04-18

**Authors:** André Toussaint, Anne Richter, Frederick Mantel, John C. Flickinger, Inga Siiner Grills, Neelam Tyagi, Arjun Sahgal, Daniel Letourneau, Jason P. Sheehan, David J. Schlesinger, Peter Carlos Gerszten, Matthias Guckenberger

**Affiliations:** Department of Radiation Oncology, University of Wuerzburg, Wuerzburg, Germany; Departments of Neurological Surgery and Radiation Oncology, University of Pittsburgh School of Medicine, Pittsburgh, PA USA; William Beaumont Hospital, Royal Oak, MI USA; Department of Radiation Oncology, Sunnybrook Odette Cancer Center, University of Toronto, Toronto, ON Canada; Princess Margaret Cancer Center, Toronto, ON Canada; University of Virginia School of Medicine, Charlottesville, VA USA; Division of Radiation Oncology, University Hospital Zurich, Zurich, Switzerland

**Keywords:** Spine radiosurgery, Vertebral metastases, Delineation, Planning variability

## Abstract

**Background:**

The aim of this study was to quantify the variability in spinal radiosurgery (SRS) planning practices between five international institutions, all member of the Elekta Spine Radiosurgery Research Consortium.

**Methods:**

Four institutions provided one representative patient case each consisting of the medical history, CT and MR imaging. A step-wise planning approach was used where, after each planning step a consensus was generated that formed the basis for the next planning step. This allowed independent analysis of all planning steps of CT-MR image registration, GTV definition, CTV definition, PTV definition and SRS treatment planning. In addition, each institution generated one additional SRS plan for each case based on intra-institutional image registration and contouring, independent of consensus results.

**Results:**

Averaged over the four cases, image registration variability ranged between translational 1.1 mm and 2.4 mm and rotational 1.1° and 2.0° in all three directions. GTV delineation variability was 1.5 mm in axial and 1.6 mm in longitudinal direction averaged for the four cases. CTV delineation variability was 0.8 mm in axial and 1.2 mm in longitudinal direction. CTV-to-PTV margins ranged between 0 mm and 2 mm according to institutional protocol. Delineation variability was 1 mm in axial directions for the spinal cord. Average PTV coverage for a single fraction18 Gy prescription was 87 ± 5 %; D_min_ to the PTV was 7.5 ± 1.8 Gy averaged over all cases and institutions. Average D_max_ to the PRV_SC (spinal cord + 1 mm) was 10.5 ± 1.6 Gy and the average Paddick conformity index was 0.69 ± 0.06.

**Conclusions:**

Results of this study reflect the variability in current practice of spine radiosurgery in large and highly experienced academic centers. Despite close methodical agreement in the daily workflow, clinically significant variability in all steps of the treatment planning process was demonstrated. This may translate into differences in patient clinical outcome and highlights the need for consensus and established delineation and planning criteria.

## Introduction

Cancer patients with systemic tumor spread frequently develop skeletal metastases. Many of these occur within the spine [1–3]. Radiotherapy (RT) is an established and recommended component of the multidisciplinary treatment of spine metastases with regard to prevention of pathologic fractures or neurological deficits and pain palliation [4–6]. Conventional RT with low dose per fraction is effective but has been shown to achieve only a rather short duration of pain response of only 3–6 months (median) [7]. Improvements in systemic treatment efficacy have prolonged survival in many cancer patients. Also, validated overall survival scores have been described for patients treated with spine metastases that has enabled identification of patient subgroups with longer survival [8–10].

Spine Stereotactic Radiosurgery (SRS), sometimes also referred to as Stereotactic Body Radiation Therapy (SBRT) to the spine, has been demonstrated to result in promising long term local control and pain palliation with low toxicity rates [11–16]. The use of SRS as an alternative treatment to conventional RT has been increasing rapidly. However, there still remains significant variability and little consensus exist regarding target volume contouring and treatment planning [17–19].

The aim of this study was to quantify the variability in SRS planning practice between five experienced international centers. This planning study is based on 4 patient cases with representative tumor lesions in 1–2 vertebrae.

## Materials and methods

### Patient cases

**Fig. 1:**
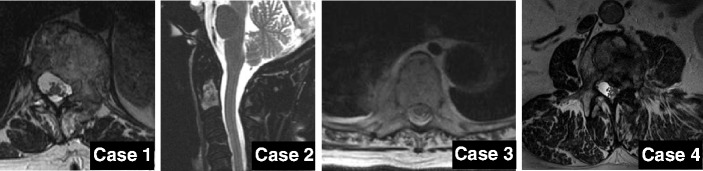
Representative slices from T2 weighted MR images of all patient cases. Case 1,3,4 are presented in axial view and case 2 in sagittal view

All centers were experienced in spine RS planning and delivery and were members of the Elekta Spine Radiosurgery Research Consortium which included University Hospital Wuerzburg, University of Pittsburgh School of Medicine, William Beaumont Hospital, Princess Margaret Hospital, University of Virginia School of Medicine. Four cases from 4 institutions were selected that covered the range of spine RS practice. The epidural extension was Bilsky score 0, 1b and 2 and paraspinal involvement was observed in 3 of 4 cases [20]. Detailed information on patient and tumor characteristics is given in Table 1. Representative images from each case are illustrated in Fig. 1.

**Table 1:** Detailed patient status parameters

Patient case:	1	2	3	4
Sex	F	F	F	M
Age	55	61	63	55
Histology:	Breast	Breast	Lung	Renal
Pain Score VAS	7	5	6	-
Paraspinal involvement:	Yes	No	Yes	Yes
Bilsky score:	1b	0	0	2
Circumferential epidural disease:	180°	90°	90°	180°
Location:	T 12-L1	C 2	T 7–8	L 3
Number of involved vertebras:	2	1	2	1

### Planning procedure

A step-wise planning procedure was performed such that variability in all stages of the planning process were analyzed independently: 1) CT-MR image registration 2) gross-tumor volume (GTV) and organ at risk (OAR) spinal cord delineation 3) clinical target volume (CTV) and planning risk volume (PRV) definition 4) planning target volume (PTV) definition 5) treatment planning based on consensus contours and 6) treatment planning based on institutional-specific contours. This was achieved by providing consensus results after each step of the planning process, forming the basis for the next step.

The planning software for treatment planning was Pinnacle^3^ in all institutions (Philips Radiation Oncology Systems, Fitchburg, WI, USA) and some institutions used additional software for image registration or delineation depending on in house protocol.

### Step 1: CT-MR image registration

The CT and MR images for each patient were electronically provided to each institution. The CT images were defined as reference image data set and the MR image as secondary image set for image fusion. The registration parameters were recorded in x (Left-Right), y (Anterior-Posterior) and z (Superior-Inferior) directions. Only rigid translations and rotations of the image sets were considered for each case.

Average registration results were calculated by taking the mean value of all registration results and were considered to be the consensus results. These consensus registration parameters were sent back to all institutions. The consensus registration parameters were then used for the following step of target definition.

### Step 2: delineation of GTV and spinal cord

GTV and spinal cord (in one case with lumbar location - thecal sac) delineation was performed according to the institution specific in-house protocols in consideration of the clinical case descriptions by all institutions. A consensus for all case specific GTV and spinal cord contours was calculated and distributed to the different institutions by the coordinating institution.

### Step 3: delineation of CTV

Delineation of CTV was based on the consensus GTV. According to step 2, a consensus for all case specific contours was calculated and distributed to the different institutions by the coordinating institution.

### Step 4: definition of PTV and PRV

PTV definition was based on the consensus CTV. The definition of the planning risk volume spinal cord (PRV_SC) was based on the consensus spinal cord (in one case with lumbar location, consensus spinal canal). Once again, a consensus for all case specific contours was calculated and distributed to the different institutions by the coordinating institution.

### Step 5: treatment planning based on consensus contours

The consensus PTV and the consensus PRV_SC were used for treatment planning. Institution specific contouring of relevant organs at risk (e.g. lungs…) and optimization guiding structures for the dose optimization process was allowed. Target volume prescription and normal tissue constraints are given below (see Step 6).

Institution specific in-house planning objectives/constraints were used for generation of the treatment plan. Both step-and-shoot IMRT as well as VMAT were allowed. All institutions used an Elekta Synergy S/Axesse equipped with the Beam Modulator MLC (4 mm leaf width) for treatment planning (Elekta Beam Modulator™, Elekta Oncology Systems, Crawley, UK). Photon energies of 6 MV and 10 MV were used for treatment planning according to individual in-house protocols and depending on tumor location. Doses were calculated with a 2 mm grid size and collapsed cone convolution algorithm.

### Step 6: treatment planning based on institutional-specific CTV contours

Treatment planning was repeated with institutional-specific GTV and CTV contours and institution specific PTV and OAR delineation by all institutions for all cases by using the identical planning objectives/constraints as in the previous planning step 5.

### Objectives and constraints for this planning study

The prescription dose to the PTV was 18 Gy in a single fraction. An attempt was made to achieve a PTV coverage of at least 90 % by the prescription dose. Coverage of 80–90 % of the target volume was acceptable. A minimum coverage of <80 % of the target volume was an unacceptable deviation. Dose inhomogeneity within the target volume was allowed.

The dose to the normal tissue was limited by considering the tolerance dose values as listed in Table 2 which was adapted from RTOG 0631 Study [21].

**Table 2:** Normal tissue constraints

Serial tissue	Volume in cm^3^	Dose Max in Gy	Endpoint (> Grade 3)
Spinal cord	<0.035	14	Myelitis
	<0.35	10	
	<1.2	7	
Cauda Equina	<0.035	16	Neuritis
	<5	14	
Sacral Plexus	<0.035	18	Neuropathy
	<5	14.4	
Esophagus^a^	<0.035	16	stenosis/fistula
	<5	11.9	
Heart/Pericardium	<0.035	22	Pericarditis
	<15	16	
Great vessels^a^	<0.035	37	Aneurysm
	<10	31	
Trachea^a^ and Larynx	<0.035	20.2	stenosis/fistula
	<4	10.5	
Skin	<0.035	26	Ulceration
	<10	23	
Stomach	<0.035	16	ulceration/fistula
	<10	11.2	
Renal hilum/vascular trunk	<2/3 volume	10.6	malignant hypertension
Parallel tissue	Critical volume in cm^3^	Dose Max	Endpoint (> Grade 3)
Lung (Right & Left)	1000	7.4	Pneumonitis
Renal cortex (Right & Left)	200	8.4	Basic renal function

^a^ Avoid circumferential irradiation

### Calculation of consensus structures

**Fig. 2:**
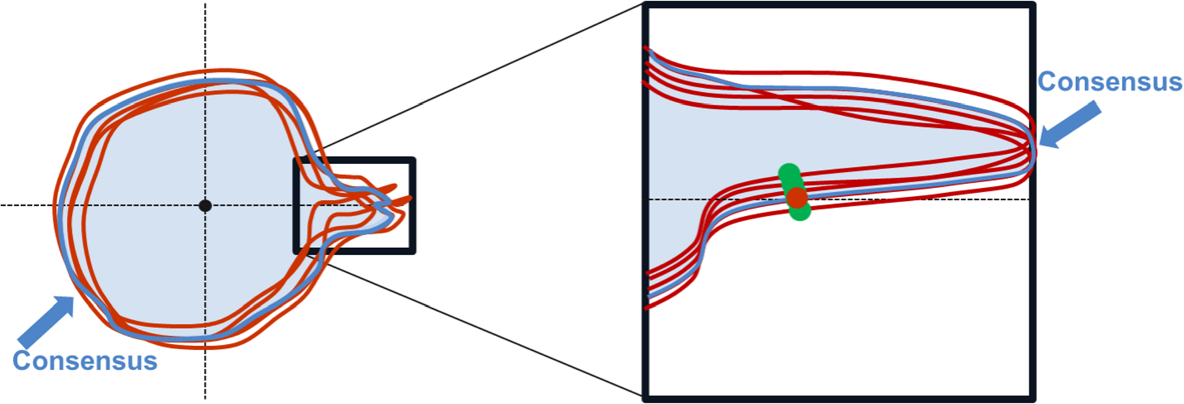
Schematic illustration of how the delineation variability was determined

For each structure GTV, CTV and SC a consensus structure was determined such that each voxel encompassed by at least two institutions was included in the consensus structure. Only one outlier was excluded (Fig. 2).

### Method for calculation of delineation variability

The consensus structure formed the basis for calculation of delineation variability. The shortest distance in each direction (Euclidean distance) was calculated between the consensus structure and the institutional structures to obtain an estimation of delineation variability for these irregular shaped contours (Fig. 2).

### Method for dosimetric plan comparison

The dose distribution of the treatment plans was evaluated by determining minimum dose (D_min_), mean dose (D_mean_), maximum dose (D_max_). The dose volume parameters according to ICRU Report 83 [22] D_98_, D_95_, D_90_, D_05_, D_02_ and V_18Gy_ were acquired for the PTV. The D_98_ of the PTV is the dose which encompasses 98 % of the PTV. Additionally, V_18Gy_ describes the part of the PTV which is treated with 18 Gy while V-Abs_18Gy_ is the total body volume treated with 18 Gy (including normal tissue). For the PRV_SC the parameters D_max_, D_0,1ccm_, V_8Gy_, V_10Gy_ were acquired.

The dose distributions were compared using the following parameters: conformity, homogeneity and target coverage, according to the RTOG recommendation [23]. Additionally, the Paddick conformity index was also determined [24]. The RTOG conformity index (CI-RTOG) represents the ratio of the volume encompassed by the prescription isodose to the target volume. Three categories of conformity index protocol compliance were defined. Plans with a conformity index value between 1.0 and 2.0 are in the normal range and are classified as not deviating from RTOG protocol. Conformity index value between 2.0 and 2.5 or between 0.9 and 1.0 are classified as having minor deviations. Value greater than 2.5 or less than 0.9 show major deviations of the dose from the RTOG protocol.

The RTOG homogeneity index (HI-RTOG) represents the ratio of maximum dose within the target volume to the prescribed dose. The normal range is up to 2.0, it indicates a minor deviation if HI-RTOG > 2.0 and a major deviation if HI-RTOG > 2.5.

The RTOG coverage (Cov-RTOG) describes the ratio between minimum dose within the target volume and the prescribed dose, it indicates a minor deviation if <0.9 and a major deviation if <0.8.

The Paddick conformity index (CI-Paddick) describes the ratio of the squared target volume covered by the prescription isodose to the arithmetic product of target volume and prescription isodose total volume. This equals the multiplication of the undertreatment ratio and the overtreatment ratio. This index has an ideal value of one and plan quality decreases with decreasing index value.

## Results

### Step 1: CT-MR image registration

The results of image registration are listed in Table 3. For each case, the standard deviation and maximum range between all institutional results were calculated.

**Table 3:** Registration variability between the five institutions

	case	X in mm	Y in mm	Z in mm	rot X in °	rot Y in °	rot Z in °
Max range	**1**	4.7	2.2	2.7	3.1	0.8	4.4
	**2**	1.7	1.1	6.5	1.2	3.7	3.8
	**3**	1.8	15.9	11.8	4.1	2.9	4.5
	**4**	2.6	2.5	3.0	12.5	2.6	3.5
	**average 1–4**	2.7	5.4	6.0	5.2	2.5	4.1
	**average 1; 2; 4**	**3.0**	**1.9**	**4.1**	**5.6**	**2.4**	**3.9**
SD	**1**	1.7	0.9	1.0	1.1	0.4	1.6
	**2**	0.7	0.4	2.5	0.5	1.7	1.4
	**3**	0.7	6.9	4.8	1.7	1.2	1.8
	**4**	1.2	1.0	1.5	4.6	1.0	1.3
	**average 1–4**	1.1	2.3	2.5	2.0	1.1	1.5
	**average 1; 2; 4**	**1.2**	**0.8**	**1.7**	**2.1**	**1.0**	**1.4**

*Abbreviations*: x (left-right), y (anterior-posterior) and z (superior-inferior) direction; SD; standard deviation; average 1; 2; 4 (bold) excludes outlier case 3

Large registration variability was observed for case 3, most likely because of low image resolution (pixelspacing 1.2 mm CT and 0.8 mm on MRI) and large slice spacing of 3 mm on CT and 4.8 mm on MR. This was a thoracic spine (T 7–8) case with MR acquired in non-treatment position that made CT-MR registration extremely challenging. Without consideration of this outlier, average registration variability ranged from 0.8 mm to 1.7 mm for translation and 1.0° to 2.1° for rotation.

### Step 2: delineation of GTV and spinal cord

The variation of the GTV contours is shown in Table 4. Variability was quantified by using the parameters SD and range in x (Left-Right), y (Anterior-Posterior) and z (Superior-Inferior) direction. On average the axial (X and Y) and longitudinal (Z) standard variation was 1.5 mm, 1.6 mm, respectively. The range was on average 3.5 mm and 3.8 mm in axial and longitudinal direction.

**Table 4:** GTV definition variability between the five institutions

	SD in mm		Range in mm	
Case	X and Y	Z	X and Y	Z
1	1.3	0.6	3.1	1.5
2	1.7	3.4	4.1	8.1
3	1.3	0.9	2.5	2.0
4	1.8	1.5	4.0	3.6
Average	1.5	1.6	3.5	3.8

For the spinal cord, the delineation variability (1 SD) between the five institutions was 1 mm in axial direction averaged over the four cases.

### Step 3: delineation of CTV and organ at risk spinal cord

Based on the consensus GTV, all institutions created a CTV which again resulted in variabilities of 0.8 mm and 1.2 mm in axial and longitudinal directions, respectively. The range was on average 1.8 mm and 2.8 mm in axial and longitudinal direction as shown in Table 5.

**Table 5:** CTV definition variability between the five institutions

	SD in mm		Range in mm	
Case	X and Y	Z	X and Y	Z
1	0.6	0.3	1.6	0.8
2	1.3	3.0	3.0	6.9
3	0.5	0.6	1.0	1.4
4	0.9	1.0	1.9	2.3
Average	0.8	1.2	1.8	2.8

### Step 4 delineation of PTV

CTV-to-PTV margins ranged between 0 mm and 2 mm according to institutional protocol [17, 25]. Margins for generation of the PRV spinal cord ranged between 1 mm and 2 mm.

### Step 5: treatment planning

All participating institutions generated one treatment plan for each case using previously generated consensus structures. The dosimetric analyses are listed in Tables 6 and 7.

**Table 6:** ICRU report parameters with average values over all cases

	PTV	
D_min_ in Gy	7.5	±1.8
D_98_ in Gy	12.2	±2.0
D_95_ in Gy	15.2	±1.7
D_90_ in Gy	17.4	±1.0
D_mean_ in Gy	19.4	±0.8
D_05_ in Gy	21.6	±1.3
D_02_ in Gy	21.9	±1.4
D_max_ in Gy	23.1	±1.6

**Table 7:** Mean of doses and volumes to PRV_SC-Consensus

	D_max_ in Gy		D_0.1ccm_ in Gy		V_8Gy_ in ccm		V_10Gy_ in ccm	
Dose to PRV_SC_consensus_ (Mean ± SD)	10.5	±1.6	9.0	±1.5	1.5	±1.6	0.1	±0.1

D_min_ to the PTV was 7.5 ± 1.8 Gy averaged over all cases and institutions. The D_90_ was 17.4 ± 1.0 Gy on average and 86.9 % ± 5.2 of the PTV was covered by the prescribed dose of 18 Gy.

D_max_ to the PRV_SC_consensus_ (spinal cord + 1 mm) was 10.5 Gy on average and variability (1 SD) of D_max_ was 1.6 Gy averaged over all cases and institutions. D_max_ in the PRV_SC was smaller than 12 Gy in 18 of 20 treatment plan trials developed by the participating institutions. For the two deviating trials, the D_0,1ccm_ was 11 Gy in maximum. The highest variability between institutions was observed in case 4, where D_max_ to the PRV_SC ranged between 6.4 Gy and 11.7 Gy. The mean absolute PRV_SC volume exposed to maximum 10 Gy was 0.1 cm^3^ with a maximum of 0.39 cm^3^.

**Fig. 3:**
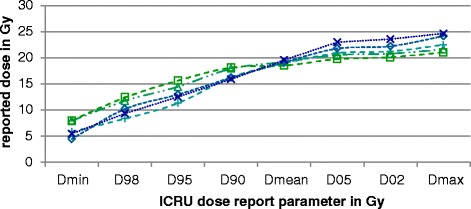
ICRU dose report parameters for one exemplary case (case 1) for all 5 participating institutions. The connecting lines should enhance the distinction between institutions

In Fig. 3 the ICRU dose report parameters are shown for one example case (case 1) for all 5 participating institutions. Good agreement is demonstrated for the parameters D_90_ and D_mean_ with a maximum deviation of 2.4 Gy and 1.1 Gy. A higher variability is demonstrated for the other dose report parameters – especially for D_98_, D_95_ and D_max_ with a maximum difference of 4.9 Gy, 5.1 Gy and 4.6 Gy for this example case. Over all cases, the maximum deviation in D_98_ and D_95_ was 8.5 Gy and 7.6 Gy

**Fig. 4:**
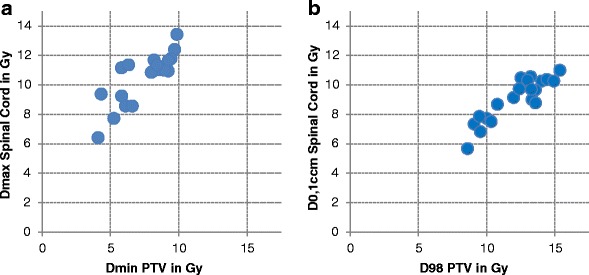
**a/b** Maximum planning risk volume spinal cord (PRV_SC) doses to PTV minimum doses and dose to 0,1ccm of spinal cord to PTV D98 for all cases and institutions

Figure 4a illustrates the maximum dose to the planning risk volume spinal cord (D_max_ PRV_SC) as a function of the minimum dose in the PTV (D_min_ PTV). Fig. 4b shows the dose to 0.1 cm^3^ of the spinal cord to the D_98_ in the PTV. A very strong correlation can be seen - especially between D_0.1ccm_(SC) and D_98_(PTV) with a coefficient of determination of R^2^ = 0.81.

For all cases and all plans, the performance parameters were calculated and analyzed. The detailed performance parameters are listed in Table 8. Cov-RTOG was 87 % on average and variability was 5 %; variability was largest in case # 1 where PTV coverage ranged between 76 and 91 %. The CI-Paddick was 0.68 on average with small variability of 0.08; the largest variability in CI-Paddick was observed in case # 2 with a range of 0.76 and 0.91 between the institutions.

**Table 8:** Mean performance parameters for all cases

Case	CI-Paddick		CI-RTOG		HI-RTOG		Cov-RTOG	
1	0.67	±0.08	1.11	±0.17	1.98	±0.34	0.86	±0.06
2	0.63	±0.08	1.19	±0.24	1.64	±0.17	0.86	±0.05
3	0.70	±0.07	1.09	±0.15	1.90	±0.31	0.87	±0.04
4	0.72	±0.05	1.10	±0.12	1.90	±0.44	0.89	±0.05
mean	0.68	±0.08	1.12	±0.18	1.86	±0.35	0.87	±0.05

One additional plan was optimized based on the PTV_individual_ of each case and institution. Doses to the consensus PTV and consensus PRV_SC were evaluated in these treatment plans to quantify the dosimetric consequences of the image-registration and contouring uncertainties.

**Fig. 5:**
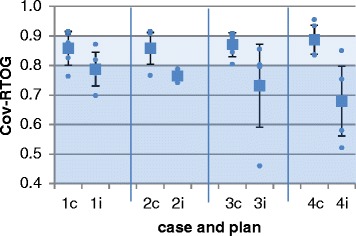
Performance parameters RTOG coverage for all analyzed plans. Abbreviation: results of case 1–4 with consensus (c) and individual (i) plans

**Fig. 6:**
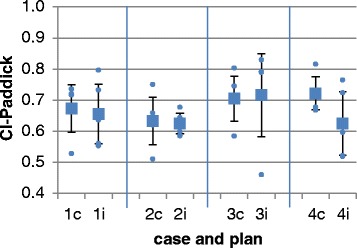
Performance parameters CI-Paddick for all analyzed plans

For the PTV_individual_ based planning process, the variations on planning performance was higher compared to the consensus based planning. Performance parameters are illustrated in Figs. 5 and 6. All discrete values are illustrated with blue dots. Mean values over all institutions are shown in blue squares with standard deviation as error bars. In Fig. 5, the minor deviation range according to the respective protocol is shaded in light blue while major deviation rang is shown in shaded deeper blue.

Cov-RTOG decreased significantly if plans were generated based on institution-individual contours (1c - 4c). The mean values of consensus plans are centered in a range that would be considered as minor deviations (<0.9) for all cases. This is contrasted with the mean of the individual plans (1i –4i), which are consistently in a range that would be considered a major deviation (<0.8) (Fig. 5).

The CI-Paddick is less affected by the image registration and delineation variability. As shown in Fig. 6, the consensus plans show on average a higher CI-Paddick and smaller deviations.

For plans on individual PTVs, the average D_min_ for PTV_consensus_ drops from 7.5 ± 1.8 Gy to 6.5 ± 0.9 Gy. The D_max_ to PRV_SC increased from 10.5 ± 1.8 Gy to 12.2 ± 2.2 Gy. Similar results were observed for D_0.1ccm_ which increased from D_0.1ccm_ 9.0 ± 1.5 Gy to 9.9 ± 1.8 Gy.

## Discussion

The aim of this work is to quantify the variability of treatment planning for spine radiosurgery in various steps of the treatment planning process between five international institutions. High variability was observed during all steps of the planning process of image registration and contouring of tumor and target volumes. For fair comparison, consensus registration parameters were determined for image fusion and consensus contours were developed for treatment plan optimization and dosimetric evaluation. Additional individual treatment plans were generated based on each individual institutions spine RS practice and showed higher variabilities in dose performance parameters for consensus target volumes and organs at risk.

For the CT-MRI registration process, a high variability was observed as reported by Ulin et al. [26]. None of the MR images were acquired in treatment position. The influence of the 3D- voxel size seems to be the main factor for registration performance. Case 1, 2 and 4 had an average voxel size for CT and MR datasets of 1.0 mm with an SD of 0.5 mm whereas case 3 had an voxel size of 4.1 mm for CT and 2.9 mm for MR dataset. Deformation of the vertebral column is well known but our results indicate that even in SBRT of a solitary vertebra, reproducible patient positioning for MR imaging should be performed to improve image registration. Additionally, all institutions explained that automatic image registration achieved unsatisfactory results and the image registration was adjusted manually. Improved software and methodologies specifically optimized for the vertebral column may therefore be required. One promising approach was suggested by Sohn et al. [27]. They present a segmental image fusion protocol which allows an improved visualization of spinal tumors and promises to achieve more consistent results. Nevertheless, image registration depends on datasets with high spatial resolution and a reproducible positioning to avoid or at least to minimize deformation.

For the delineation process, the highest variability was observed for GTV delineation, which was about 1.5 mm on average. The following step of CTV definition, which was based on the consensus GTV contour, resulted in lower variability between institutions of about 1 mm. The CTV-to-PTV margin ranged between 0 mm and 2 mm. These variabilities clearly show the need for further standardization of imaging and of delineation guidelines. Recently, GTV delineation variability was evaluated for SBRT of stage I non-small cell lung cancer and the overall delineation variability was 2.1 mm [28]. Tseng et al. results support the importance of controlling bulk patient motion and the practice of applying a planning organ-at-risk margin [29]. Several studies have investigated the inter-observer variability during the target definition of other entities. All of them agreed that the consistency of contouring can be improved by education and training, consensus guidelines and multi-institutional collaborations [30–33]. Also, a high degree of inter-observer variability was seen for brain SRS [34, 35]. Deviations between treatment planning based on individual and consensus structures may show a potential influence on patient outcome.

An overview of methods of spine radiosurgery for the participating institutions was described by Guckenberger et al. [17]. Good agreement was seen for the imaging acquisitioning techniques and safety margins concepts. However, treatment plan acceptance criteria varied substantially between all institutions. For this work, a D_90_ of 18 Gy in the PTV was suggested as parameter for plan acceptance. Even though the treatment planning was performed based on consensus contours, the acceptance criteria for D_90_ varied up to 3 Gy. This might be due to different approaches to achieve target coverage and sparing of the organs at risk, especially the spinal cord. The tolerance dose to the spinal cord is limited to a maximum dose while other institutions limit the dose to different sub-volumes (0.1 cm^3^, 0.4 cm^3^ or 0.04 cm^3^). This disagreement was already stated by Guckenberger et al. [17]. Additionally, changes/deviations in beam configuration and optimization goals can lead to different planning results. Again, these variabilities show the need of strict and consistent acceptance criteria. Similar findings were published by Esposito et. al who investigated variability in treatment planning for stereotactic radiotherapy of liver metastasis [36]. In a multicenter study, they found significant differences for target coverage and OAR sparing due to different optimization strategies selected by the planners. Another multi-institutional study evaluated dosimetric parameters for SBRT of lung lesions and how much the guidelines provided in the literature are being successfully implemented in a variety of clinics [37]. They analyzed PTV coverage and conformality of their treatment plan and found that and the conformality index of 50 % was the most difficult to meet depending on tumor size and location.

## Conclusion

Spinal radiosurgery (SRS) for vertebral metastases is a rapidly evolving treatment modality that has shown promising results in terms of pain and tumor control. However, the methodology and implementation of SRS has not yet been sufficiently standardized. Further studies are needed to establish whether the variability observed in this study will influence the clinical outcomes.
